# Diverging trends in incidence and mortality of testicular cancer in Denmark, 1943-1982.

**DOI:** 10.1038/bjc.1986.79

**Published:** 1986-04

**Authors:** A. Osterlind

## Abstract

Between 1943 and 1982, 5,140 new cases of testicular cancer were diagnosed in Denmark. The age-standardized incidence rate more than doubled in the period. Striking variations is seen in the age-relationship over time with a four-fold increase in incidence for men aged 15-24 years while no increment was observed for those above 65 years of age. The increase with time in the risk could be accounted for by a cohort effect. The mortality rate did not parallel the incidence rate and a 50% decrease in mortality rate appeared in the period 1978-1982. Introduction of combination chemotherapy including cis-platinum is one of the main factors responsible for this beneficial development. The observed pattern of diverging trends in incidence and mortality of testicular cancer implies that mortality rates do not reflect the incidence and will accordingly be unsuited as a basis for aetiological considerations based on trends. The brisk increase in the risk of testicular cancer, especially among young men is in accordance with trends in other western countries, and prompts an enforced search for suspected or new risk factors.


*Br. J. Cancer* (1986), **53**, 501–505

### Diverging trends in incidence and mortality of testicular cancer in Denmark, 1943–1982

A. Østerlind

*The Danish Cancer Registry, Institute of Cancer Epidemiology under the Danish Cancer Society, Landskronagade 66, 4th floor, DK-2100 Copenhagen Ø.*

**Summary** Between 1943 and 1982, 5,140 new cases of testicular cancer were diagnosed in Denmark. The age-standardized incidence rate more than doubled in the period. Striking variation is seen in the age-relationship over time with a four-fold increase in incidence for men aged 15–24 years while no increment was observed for those above 65 years of age. The increase with time in the risk could be accounted for by a cohort effect. The mortality rate did not parallel the incidence rate and a 50% decrease in mortality rate appeared in the period 1978–1982. Introduction of combination chemotherapy including cis-platinum is one of the main factors responsible for this beneficial development. The observed pattern of diverging trends in incidence and mortality of testicular cancer implies that mortality rates do not reflect the incidence and will accordingly be unsuited as a basis for aetiological considerations based on trends. The brisk increase in the risk of testicular cancer, especially among young men is in accordance with trends in other western countries, and prompts an enforced search for suspected or new risk factors.

Denmark has the highest reported national age-standardized incidence rates of testicular cancer in the world (Waterhouse *et al.*, 1982), even though the disease only accounts for 1.8% of all cancers among Danish men. It is the most common malignancy among men aged 15–44 years, in whom it accounts for 23% of all cancers.

Cancer of the testis thus differs from other cancers in being a disease of young adults and 50% of the cases are diagnosed before the age of 35 years in contrast to cancers of all sites of which 50% are diagnosed after the age of 70 years.

In view of the substantial improvement in survival in recent years related to chemotherapy with cis-platinum, bleomycin, vinblastine and etoposide (von der Maase *et al.*, 1984; Peckham *et al.*, 1985) the present paper draws attention to the divergent trends in incidence and mortality of testicular cancer in Denmark, where such information is available for a well-defined national population for a longer period than anywhere else in the world. Other differences between incidence and mortality of the disease will also be highlighted.

### Materials and methods

All cases of cancer occurring in the entire Danish population are reported to the Danish Cancer Registry, founded in 1942. The registration system is based on notifications from all clinical departments of Danish hospitals and practicing physicians, and supplemented with information from death certificates. Recent evaluations indicate that the registration may for all practical purposes be regarded as complete and valid (Østerlind & Jensen, 1985). All new cases of cancer since 1943 have been categorized by trained coders according to an extended version of the Seventh Revision of the International Classification of Disease (1957). Additional codes enable a division by morphologic type. For the period 1978–82 cases have also been categorized according to the International Classification of Disease for Oncology (ICD-O) (1976) which includes a detailed description of morphological types.

All incident cases of cancer originating in the testis are included. The diagnosis was histologically verified in 98% of the cases in the decade of 1973–1982 compared with 91% in the period 1943–1952 (Clemmesen, 1965; Danish Cancer Registry, 1982; Danish Cancer Registry, 1983). Data for the years 1981 and 1982 are preliminary as they have not yet been supplemented with information from death certificates. The lack is of minor importance, however, as less than 1/2% of the registered testicular cancer cases derive from death certificates only (Danish Cancer Registry, 1983).

The number of deaths per year in persons with a certified diagnosis of testicular cancer is available for the whole period 1943–1982 (Causes of Death in the Kingdom of Denmark, 1940–1982).

All incidence and mortality rates are average annual rates per 100,000 men, with the Danish male population at the midyear of the period as denominator. Age is standardized by the direct

Received 24 October 1985.

© The Macmillan Press Ltd., 1986

method to the World Standard population (Waterhouse *et al.*, 1982).

### Results

A total of 5,140 cases were diagnosed with cancer of the testis in Denmark in the years 1943 to 1982. During this period the annual number of incident cases tripled from around 70 to 220 (Table I). The age-standardized incidence rate has been rising steadily, by ~4% annually and has more than doubled in the period (Table I).

Figure 1 shows the annual average age-specific incidence rates for the three 5-year periods 1943–1947, 1968–1972 and 1978–1982. Cancer of the testis is very rare in children especially in the age-group 3–14 years. Sixty-five percent of the testicular cancers in childhood are diagnosed before the age of 3 years. The age-specific incidence curve shows a small peak in infancy that tapers off after the age of 4. At puberty the curve rises sharply to take a bimodal course with a prominent peak at age 20 to 45 and a lesser peak after the age of 70 years. A comparison of the age-specific incidence rates reveals that there has been quite a different trend in the various age-groups. No increase has occurred in children (aged 0–14 years) during the 40 years period. Among younger men aged 15–24 years the incidence rate increased 4 times since 1943 whereas the incidence showed a 2–3 fold increase for men aged 25–64 years. For those over 65 years of age the incidence remained constant or decreased slightly. Examination of the data by birth cohorts discloses a trend (Figure 2) toward higher rates before the age of 60 years for the cohorts 1918 to 1958, whereas no increase is seen for the earlier cohorts representing the older age-groups. (For the sake of clarity every second birth cohort curve is omitted.)

**Figure 1** Age-specific incidence rates of testicular cancer in Denmark; (—) 1943–1947; (--) 1968–1972 and (-·-) 1978–1982.

An analysis of the incidence rates for the capital, the provincial towns and the rural areas (Figure 3) shows that the risk of testicular cancer has increased in about the same degree in the different geographical areas.

The rise in the incidence affects seminomas and non-seminomas to the same degree. During the entire period seminomas accounted for some 52% of the testicular cancers whereas non-seminomas rose from 33% in 1943–1947 to 44% in 1978–1982 (Table II).

*Table I* Incidence of and mortality from testicular cancer in Denmark, 1943–1982

| *Time period* | *Number of cases per year* | *Age-standardized incidence rate per 100,000*[a] | *Number of deaths per year* | *Age-standardized mortality rate per 100,000*[a] | *Ratio mortality rate/ incidence rate* |
|---|---|---|---|---|---|
| 1943–1947 | 69 | 3.1 | 28 | — | — |
| 1948–1952 | 76 | 3.3 | 29 | — | — |
| 1953–1957 | 90 | 3.9 | 41 | 1.7 | 0.43 |
| 1958–1962 | 106 | 4.6 | 45 | 1.9 | 0.41 |
| 1963–1967 | 122 | 5.0 | 52 | 2.1 | 0.42 |
| 1968–1972 | 153 | 6.0 | 54 | 2.1 | 0.26 |
| 1973–1977 | 192 | 7.2 | 52 | 1.9 | 0.26 |
| 1978–1982 | 219 | 8.0 | 28 | 1.0 | 0.13 |

[a]World Standard.

**Table II** Number (and percent) of testicular cancer grouped according to histological type and time period, 1943–1982

| *Time period* | *Seminomas* | *Non-seminomas* | *Other and unspecified* | *Sarcomas* | *Total* |
|---|---|---|---|---|---|
| 1943–1947 | 182 (53) | 115 (33) | 44 (13) | 4 (1) | 345 |
| 1948–1952 | 220 (58) | 122 (32) | 32 (8) | 7 (2) | 381 |
| 1953–1957 | 260 (58) | 148 (33) | 35 (8) | 7 (1) | 450 |
| 1958–1962 | 292 (55) | 211 (40) | 19 (4) | 8 (1) | 530 |
| 1963–1967 | 307 (50) | 266 (44) | 23 (4) | 15 (2) | 611 |
| 1968–1972 | 346 (45) | 359 (47) | 33 (4) | 27 (4) | 765 |
| 1973–1977 | 488 (51) | 410 (43) | 35 (3) | 29 (3) | 962 |
| 1978–1982 | 574 (52) | 476 (44) | 32 (3) | 14 (1) | 1,096 |
| Total | 2,669 (52) | 2,107 (41) | 253 (5) | 111 (2) | 5,140 |

**Figure 2** Age-specific incidence rates of testicular cancer for birth cohorts born between 1873 and 1962.

**Figure 3** Age-standardized (World Standard) average annual incidence rates of testicular cancer in various parts in Denmark, 1943–1977. (—) Capital; (--) suburbs; (-·-) provincial towns; (····) rural.

During the period 1943–1947 the average annual number of deaths was 28 or exactly the same number as in the most recent time period. The number has however, not been constant during the period (Table I). The age-standardized mortality rate rose slightly from 1.7 to 2.1 per 100,000 during the period 1953–1967, stabilized until a decrease of 50% appeared in the period 1978–1982 from 1.9 to 1.0 per 100,000 (Table I, Figure 4). The ratio of the age-standardized death rates to the age-standardized incidence rates was 0.43 at the beginning of the period, but as a result of the increasing incidence rate and the decreasing mortality rate the ratio now is only 0.13 (Table I).

The age-specific mortality rates for the two 5-year periods 1968–1972 and 1978–1982 show the recent decrease in the mortality (Figure 5). The curves are bimodal like the incidence curves but the mortality rates for men aged 70 years or more are similar to the rates for men aged 25–40. When comparing the two mortality curves for the two periods it is apparent that the distance between them is similar for all age-groups except for men aged 25–29 years which may be accidental. This

**Figure 4** Age-standardized (World Standard) average annual incidence rates (○) and mortality rates (●) of testicular cancer in Denmark, 1943–1982.

**Figure 5** Age-specific mortality rates of testicular cancer in Denmark, 1968–1972 (—) and 1978–1982 (---).

implies that a decrease in mortality rates has taken place for all age-groups.

### Discussion

In 1969 Clemmesen found that the incidence of testicular cancer was increasing in Denmark. The present findings corroborate those previously reported data and show that the incidence continues to rise. Risk has more than doubled during the observation period (Table I) and 1 out of 200 men in Denmark is now likely to develop testicular cancer before the age of 50 years. A similar increasing trend is seen in other western countries although at lower levels.

It is unlikely that a change in histopathologic criteria or a more intense diagnostic activity has contributed to any great extent to this trend during the period of study. The pronounced increase appeared among young men in whom the diagnostic activity must be regarded as optimal during the entire period while the incidence for men aged 65 years or more was constant. These facts seem to contradict major changes in the diagnostic activity. With regard to the histopathologic criteria there have been some minor changes in the subtyping of the non-seminoma germ cell tumours, but there has been no inclusion of tumour types which previously were not regarded as testicular cancer. Carcinoma in situ is not included in the Cancer Registry material.

During the entire period seminomas accounted for some 52% of the testicular cancers whereas non-seminomas rose from 33% in 1943–1947 to 44% in 1978–1982. The changing ratio for non-seminoma is likely to be explained by a parallel reduction in tumours with unspecified histology. During the period 1978–1982 germ cell tumours accounted for 96% of the testicular cancers and of these 55% were seminomas and 45% were non-seminomas. The same proportion between the two histological groups has been observed elsewhere (Nethersell *et al.*, 1984; Teppo, 1983).

The increase in incidence of testicular cancer is particularly pronounced among young men as also seen elsewhere. The fact was not yet evident at the time of a previous examination of the Danish figures (Clemmesen, 1969) but the changes are now clearly reflected from the patterns of the birth cohort curves which reflect a steady increase in incidence for the age-groups below 60 years of age since the cohort born in the period 1913–1922.

The data enabled separate analysis of the incidence rates for the capital, the provincial towns and the rural areas. The incidence rates were similar in all the geographical areas in the early forties, but in the period 1958–1962 the rate for the capital had doubled compared to the rural areas where the incidence rate remained rather constant (Clemmesen, 1969). This difference has almost disappeared during the last 10–15 years and now the risk for testicular cancer is only some 15% less in the rural areas compared to the urban areas.

A rising mortality rate from testicular cancer as observed in Denmark during the period 1953–1967 has also been reported from other western countries (Petersen & Lee, 1972; Davies, 1981). The mortality rate in Denmark has not increased parallel to the incidence rate and in contrast to most other cancers the mortality rate has actually decreased by 50% during the latest decade (from 1973–1977 to 1978–1982). It is possible that the decreases relate more to certain types of testicular tumour than others, but the use of mortality data do not permit distinction of different histologies. This reduction is most likely a result of improved treatment, especially achieved after introduction of combination chemotherapy including cis-platinum. Reports from other countries regarding mortality data from the last decade are not yet available. A similar trend of decreasing mortality rates would be expected, however, as the new effective chemotherapy regimens presumably have been introduced almost everywhere.

Similar diverging trends in incidence and mortality of testicular cancer are likely to occur in other countries as well. As mortality is a composite of incidence and survival, the improved survival of patients with testicular cancer implies that mortality rates do not reflect the incidence and they will accordingly be unsuited as a basis for aetiological considerations based on trends. The changing incidence rate in particular among young men and the higher risk among recent cohorts together with international differences seems to indicate that new risk factors have arisen or that the influence from previous factors has intensified. This development of the disease suggests that a large fraction of the current testicular cancer are potentially preventable.

We thank Peter Brown for help in processing the data, Ms Aase Larsen for drawing the figures and Ms Vivi Clemmensen for manuscript preparation.

The Danish Cancer Registry is a Research Institute under the Danish Cancer Society.

### References

CLEMMESEN, J. (1965). Statistical studies in the aetiology of malignant neoplasms, vol. I. *Acta Pathol. Microbiol. Scand.*, Suppl. **174**, 45.

CLEMMESEN, J. (1969). Statistical studies in the aetiology of malignant neoplasms, vol. III. *Acta Pathol. Microbiol. Scand.*, Suppl. **209**, XV–XLIII.

*Causes of Death in the Kingdom of Denmark, 1940–1982.* The National Board of Health: Copenhagen. (In Danish.)

DANISH CANCER REGISTRY (1983). *Cancer Incidence in Denmark 1978, 1979 and 1980.* Danish Cancer Registry: Copenhagen.

DANISH CANCER REGISTRY (1982). *Incidence of Cancer in Denmark, 1973–1977.* Danish Cancer Registry: Copenhagen.

DAVIES, J.M. (1981). Testicular cancer in England and Wales: Some epidemiological aspects. *Lancet*, **i**, 928.

NETHERSELL, A.B.W., DRAKE, L.K. & SIKORA, K.S. (1984). The increasing of testicular cancer in East Anglia. *Br. J. Cancer*, **50**, 377.

PECKHAM, M.J., HORWICH, A., BLACKMORE, C. & HENDRY, W.F. (1985). Etoposide and cisplatin with or without bleomycin as first-line chemotherapy for patients with small-volume metastases of testicular nonseminoma. *Cancer Treatment Rep.*, **69**, 483.

PETERSEN, G.R. & LEE, J.A.H. (1972). Secular trends of malignant tumors of the testis in white men. *J. Natl. Cancer Inst.*, **49**, 339.

TEPPO, L. (1983). Histology of testicular cancer in Finland. In *An International Survey of Distributions of Histologic Types of Tumours of the Testis and Ovary*, Stalsberg, (ed.) vol. 75, p. 81. UICC Technical Report Series: Geneva.

VON DER MAASE, H., ENGELHOLM, S.A. & RØRTH, M. (1984). Non-seminomatous testicular germ cell tumours in Denmark 1976–1980. *Acta Radiol. Oncol.*, **23**, Fasc. 4, 255.

WATERHOUSE, J., MUIR, C.S., SHANMUGARATNAM, K. & POWELL, I. (eds) (1982). *Cancer Incidence in Five Continents*, **IV**. IARC Sci. Publ. no. 42. IACR: Lyon.

WORLD HEALTH ORGANIZATION (1957). *Manual of the International Statistical Classification of Diseases, Injuries, and Causes of Death. 1955 Revision (7th).* WHO: Geneva.

WORLD HEALTH ORGANIZATION (1976). *International Classification of Diseases for Oncology (ICD-O).* First edition. WHO: Geneva.

ØSTERLIND, A. & MØLLER JENSEN, O. (1985). Evaluation of registration of cancer cases in Denmark in 1977. Preliminary evaluation of registration of cancer cases by the Cancer Registry and the National Patient Registry. *Ugeskr. Laeger*, **147**, 2483. (In Danish).


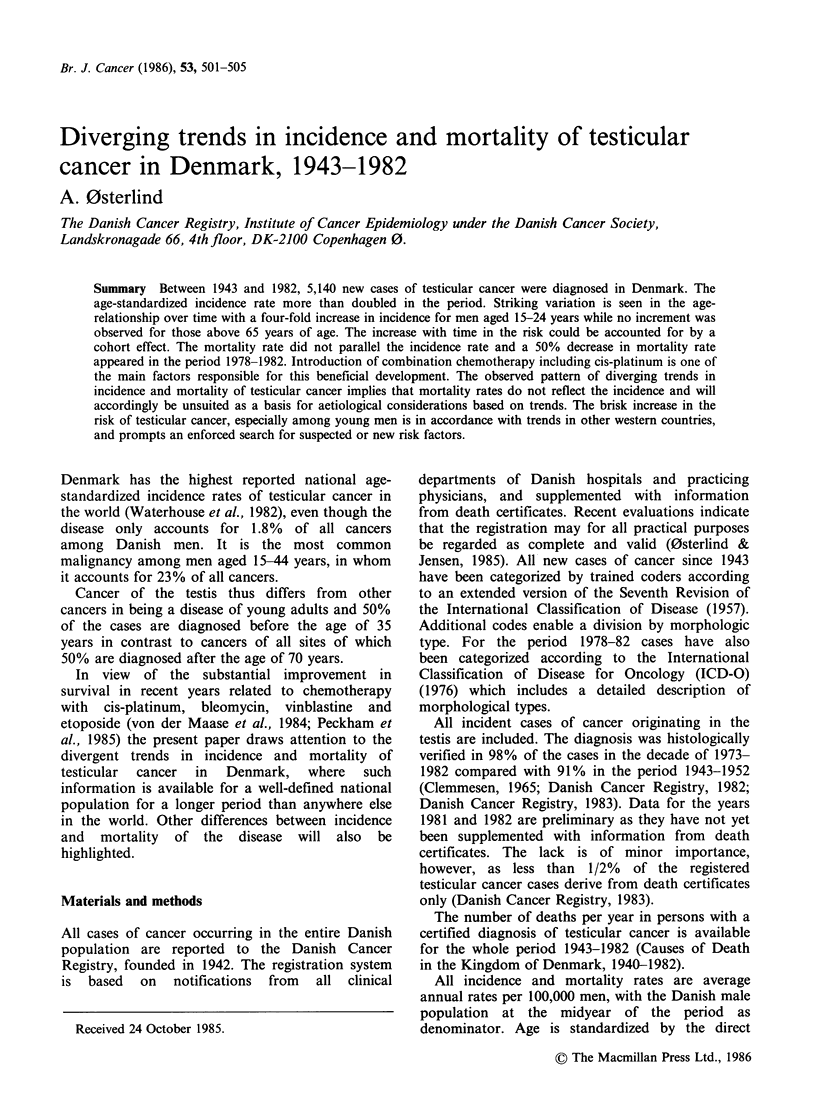

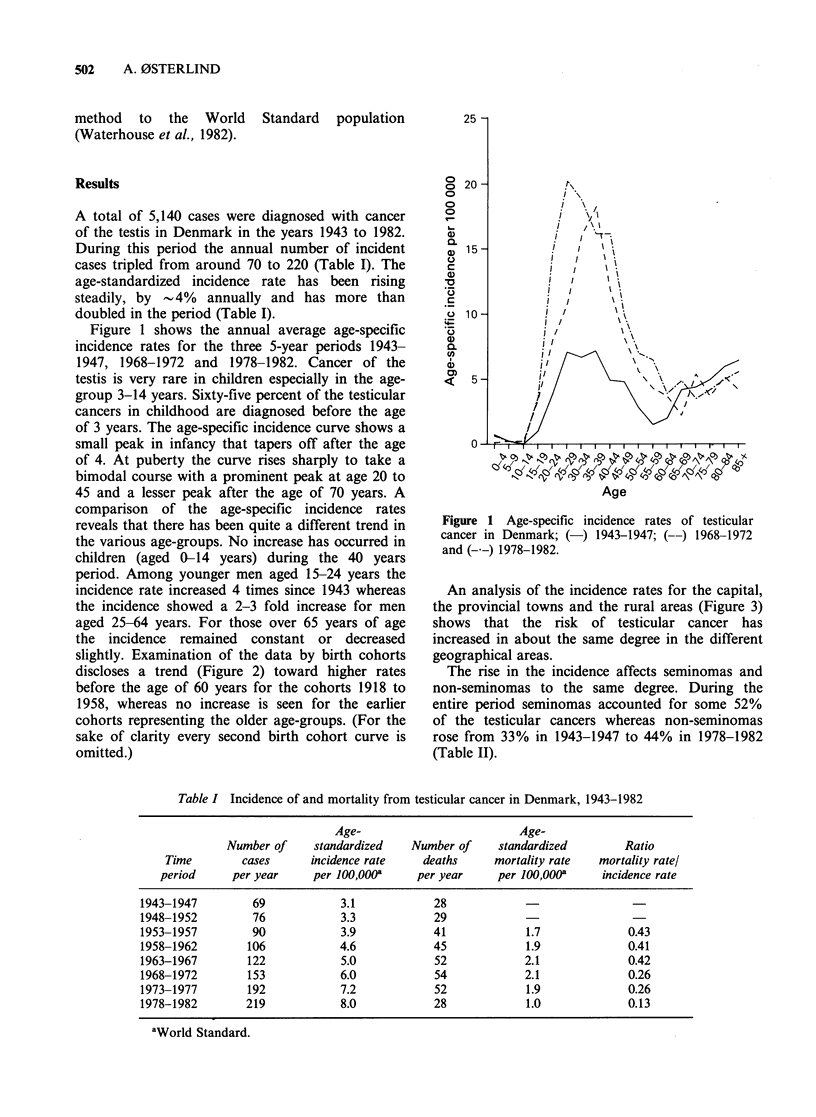

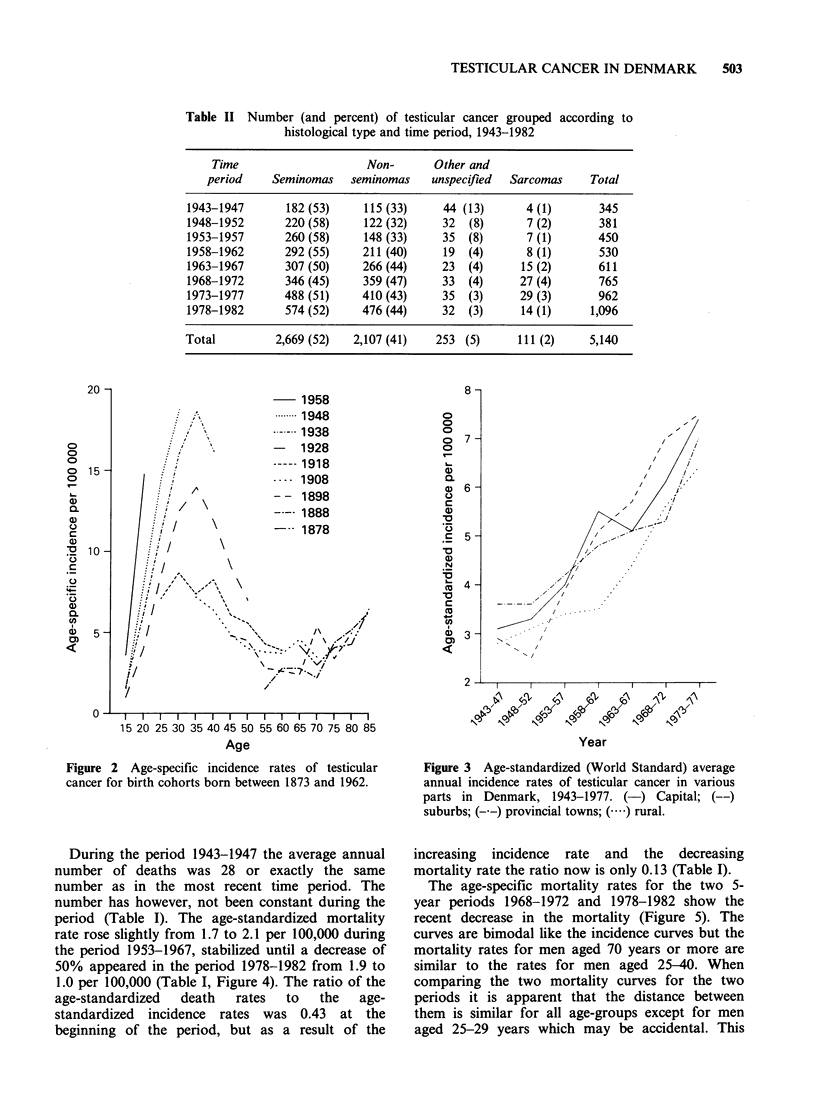

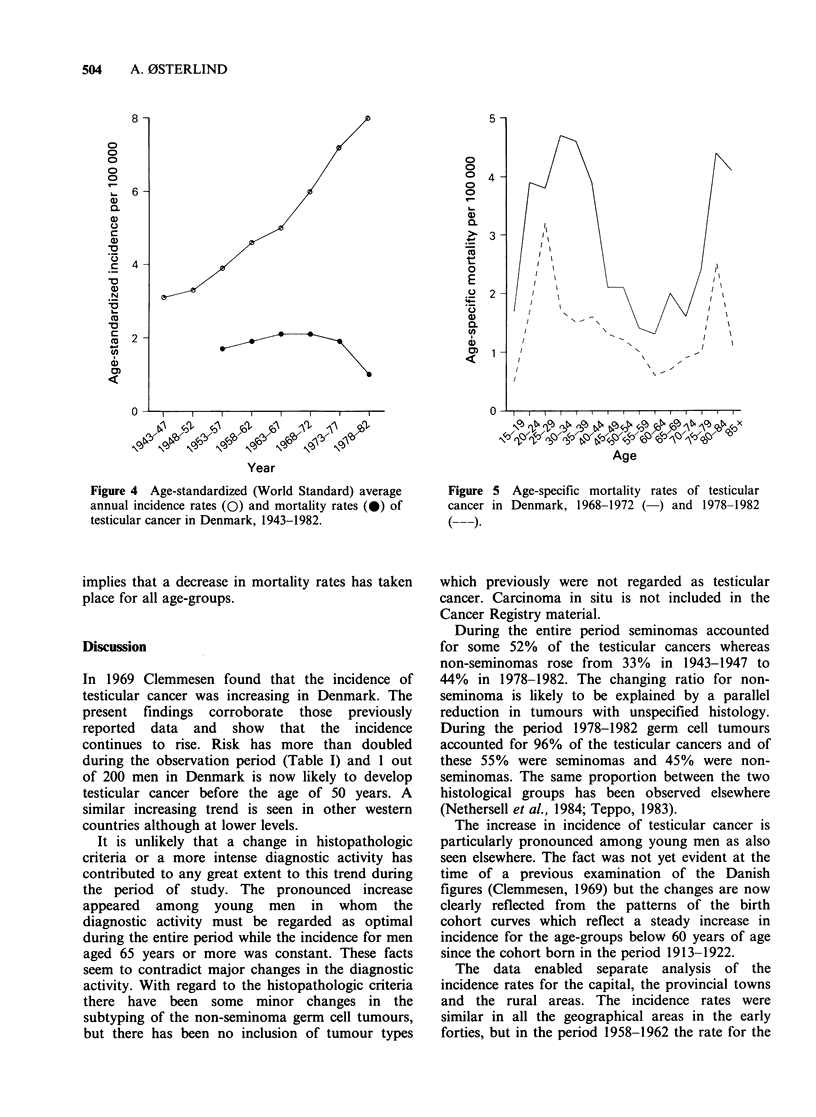

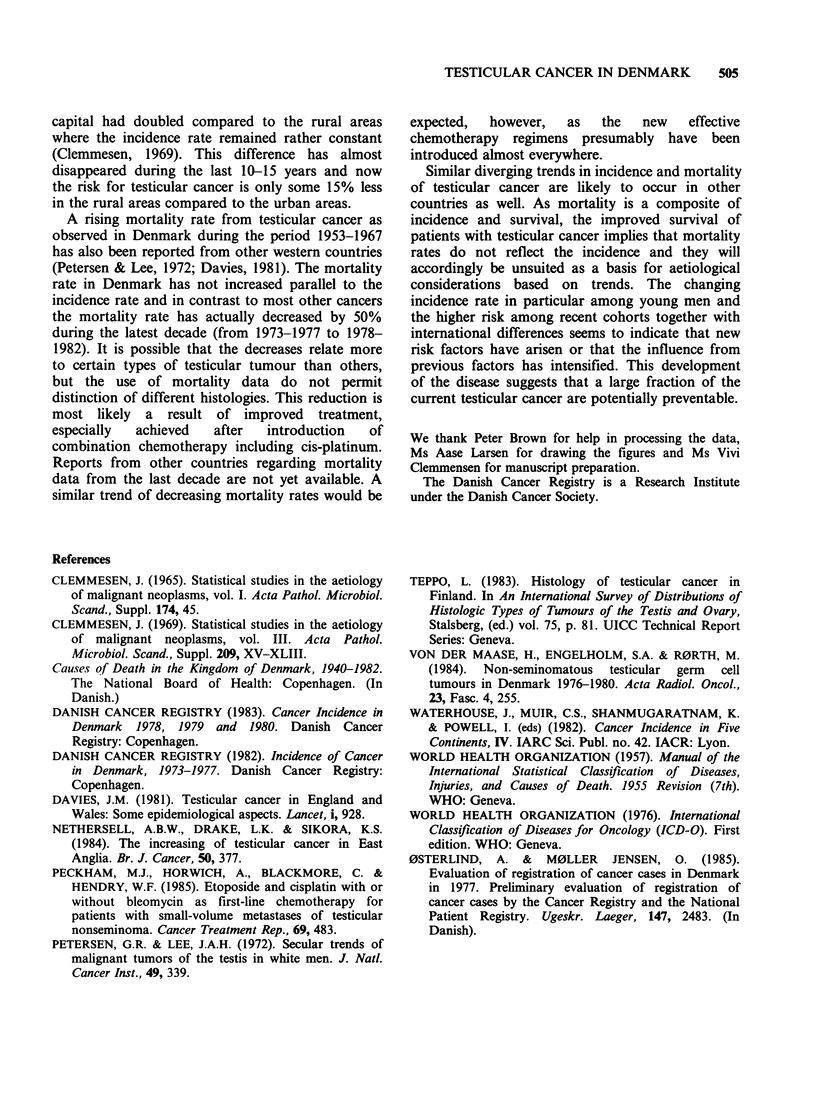


## References

[OCR_00570] Clemmesen J. (1969). Statistical studies in the aetiology of malignant neoplasms. 3.. Acta Pathol Microbiol Scand Suppl.

[OCR_00588] Davies J. M. (1981). Testicular cancer in England and Wales: some epidemiological aspects.. Lancet.

[OCR_00592] Nethersell A. B., Drake L. K., Sikora K. (1984). The increasing incidence of testicular cancer in East Anglia.. Br J Cancer.

[OCR_00597] Peckham M. J., Horwich A., Blackmore C., Hendry W. F. (1985). Etoposide and cisplatin with or without bleomycin as first-line chemotherapy for patients with small-volume metastases of testicular nonseminoma.. Cancer Treat Rep.

[OCR_00604] Petersen G. R., Lee J. A. (1972). Secular trends of malignant tumors of the testis in white men.. J Natl Cancer Inst.

[OCR_00618] von der Maase H., Engelholm S. A., Rørth M., Sandberg Nielsen E., Schultz H. P., Svennekjaer I. L., Vaeth M. (1984). Non-seminomatous testicular germ cell tumours in Denmark 1976-1980. Results of treatment.. Acta Radiol Oncol.

